# Preoperative prediction of cervical cancer survival using a high-resolution MRI-based radiomics nomogram

**DOI:** 10.1186/s12880-023-01111-5

**Published:** 2023-10-11

**Authors:** Jia Li, Hao Zhou, Xiaofei Lu, Yiren Wang, Haowen Pang, Daniel Cesar, Aiai Liu, Ping Zhou

**Affiliations:** 1 Department of Radiology, The Affiliated Hospital of Southwest Medical University, Luzhou, China; 2 Department of Cardiology, West China Hospital, Sichuan University/West China School of Nursing, Sichuan University, Chengdu, China; 3 School of Nursing, Southwest Medical University, Luzhou, China; 4 Department of Oncology, The Affiliated Hospital of Southwest Medical University, Luzhou, China; 5 Department of Gynecology Oncology, National Cancer Institute, Rio de Janeiro, Brazil

**Keywords:** Cervical cancer, Prediction, Nomogram, Magnetic resonance imaging (MRI)

## Abstract

**Background:**

Cervical cancer patients receiving radiotherapy and chemotherapy require accurate survival prediction methods. The objective of this study was to develop a prognostic analysis model based on a radiomics score to predict overall survival (OS) in cervical cancer patients.

**Methods:**

Predictive models were developed using data from 62 cervical cancer patients who underwent radical hysterectomy between June 2020 and June 2021. Radiological features were extracted from T2-weighted (T2W), T1-weighted (T1W), and diffusion-weighted (DW) magnetic resonance images prior to treatment. We obtained the radiomics score (rad-score) using least absolute shrinkage and selection operator (LASSO) regression and Cox’s proportional hazard model. We divided the patients into low- and high-risk groups according to the critical rad-score value, and generated a nomogram incorporating radiological features. We evaluated the model’s prediction performance using area under the receiver operating characteristic (ROC) curve (AUC) and classified the participants into high- and low-risk groups based on radiological characteristics.

**Results:**

The 62 patients were divided into high-risk (*n* = 43) and low-risk (*n* = 19) groups based on the rad-score. Four feature parameters were selected via dimensionality reduction, and the scores were calculated after modeling. The AUC values of ROC curves for prediction of 3- and 5-year OS using the model were 0.84 and 0.93, respectively.

**Conclusion:**

Our nomogram incorporating a combination of radiological features demonstrated good performance in predicting cervical cancer OS. This study highlights the potential of radiomics analysis in improving survival prediction for cervical cancer patients. However, further studies on a larger scale and external validation cohorts are necessary to validate its potential clinical utility.

## Background

Cervical cancer is a leading cause of cancer-related deaths in women globally [1] and a significant public health concern in developing countries [2]. Early detection and treatment are crucial [3, 4]. Surgery, combined with standard treatments such as radiotherapy and chemotherapy, has potential therapeutic value [5–7]. Unfortunately, approximately 1 in 3 patients will experience varying degrees of cancer recurrence [8], and the overall prognosis has not improved significantly. In the 2018 edition of the International Federation of Gynecology and Obstetrics (FIGO) staging system for cervical cancer, imaging examination plays a central role [9]. The revised guidelines state that cervical cancer is best evaluated through imaging, and individual imaging or pathological analysis can be used to stage the cancer. Magnetic resonance imaging (MRI) is currently the standard technique for local staging and prognostic evaluation of cervical cancer before treatment [10–13]. MRI is effective in determining the cancer stage for treatment optimization by evaluating parauterine invasion, tumor subtype and grade, and lymph node metastasis (LNM). Additionally, it can predict tumor recurrence.

Several studies have utilized MRI to predict parametrial invasion before surgery, with T2-weighted imaging (T2WI) and a combined analysis with diffusion-weighted imaging (DWI) considered effective tools to rule out invasion [14]. In recent years, radiomics has become increasingly utilized in medical fields and has been reported to improve the accuracy of tumor diagnosis and treatment response assessment by providing high-dimensional features extracted from digital medical data in a noninvasive and cost-effective manner [15, 16]. Radiomics can also provide potential predictive information on histological tumor differentiation, LNM, and lymphovascular lumen invasion in cervical cancer [17–19]. However, to our knowledge, no published studies have focused on the predictive ability of a combined radiology model [T1-weighted imaging (T1WI), T2WI, and DWI] on cervical cancer survival.

Therefore, the aim of this study is to validate radiological features with clinical value in predicting the overall survival (OS) of cervical cancer patients who underwent surgery in combination with radiotherapy and chemotherapy.

## Methods

We included 62 patients with cervical cancer who were treated between June 2020 and June 2021 at the Affiliated Hospital of Southwest Medical University, Luzhou City, Sichuan Province, China. We extracted and analyzed all patient data in accordance with the Declaration of Helsinki, and obtained ethical approval from the Ethics Committee of the Affiliated Hospital of Southwest Medical University for the retrospective data analysis. Cervical cancer diagnosis was based on FIGO (2018 edition) guidelines. To be eligible for the study, patients had to meet the following criteria: (I) confirmed diagnosis of cervical cancer by pathology between IB and IVA based on the FIGO 2018 staging; (II) underwent enhanced pelvic MRI within 2 weeks before treatment; and (III) underwent radical hysterectomy and bilateral pelvic lymph node dissection or combined radiotherapy and chemotherapy. Patients were excluded if they met any of the following criteria: (I) received neoadjuvant chemotherapy or radiotherapy; (II) diagnosed with other simultaneous cancer; or (III) lacked clinical data.

All patients received external beam radiation therapy (EBRT) [95% of the planned target volume (PTV) 50.4 Gy/28 times] and high-dose rate intracavitary radiotherapy (HDR-ICR) (30–36 Gy/5–6 times) in conjunction with weekly chemotherapy. The end time of radiotherapy was taken as the reference time point, and OS was defined as the interval from the date of treatment to the date of cancer-related death.

### Image acquisition

MRI scans were performed using a 3.0 T MR scanner (Achieva 3.0 T, Philips Healthcare, Hamburg Germany; MAGNETOM Prisma, Siemens Healthineers, Erlangen, Germany) before treatment. No patients had an absolute contraindication to MRI.

The scan range was set to encompass the whole pelvis and the scan location line was consistent. The scan sequence included non-fat suppression T1WI, non-fat suppression T2WI, and DWI. The main scan parameters were as follows: (I)T1WI images:repetition time/echo time (TR/TE), 600/11 ms;field of view (FOV), 250 × 218 mm;the number of excitations (NEX), 2;flip angle,150;pixels spacing,0.390 × 0.390 mm;slice thickness, 4 mm;spacing between slices,4.8 mm;acquisition matrix, 640–560. (II)T2WI cross-section: fast rotation echo [fast spin echo (FSE)]-XL, echo time (TE) 80 ms, repetition time (TR) 5,182 ms (average), slice thickness 5 mm, layer interval 1 mm, field of view (FOV) 26–30 cm, matrix 352 × 320; sagittal plane: FSE-XL, TE 102 ms, TR 4,138 ms (average), layer thickness 4 mm, layer interval 0.4 mm, FOV 24–32 cm, matrix 320 × 288; and (III) DWI cross-section: a single-shot echo planar imaging (EPI) sequence, TE minimum, TR 3,500 ms, FOV 26–30 cm, matrix 160 × 160 mm, layer thickness 8 mm, interlayer 1 mm, excitation times [number of excitations (NEX)] 4, diffusion-sensitive gradient b = 0 s/mm^2^ and b = 1,000 s/mm^2^.

The original images were transferred to the postprocessing workstation (GE 4.5 Workstation), and apparent diffusion coefficients (ADCs) were reconstructed using FuncTool software. For region of interest (ROI) drawings, all images were transferred into 3D Slicer software. An MRI diagnostic physician (with 4 years of experience) determined the tumor location and boundary on DWI according to each sequence image and drew all the tumor areas layer by layer. All ROIs were individually confirmed or revised by a deputy chief physician with more than 10 years’ experience in gynecological MRI diagnosis. The position of the ROI drawn on the DWI was matched with the ADC.

For image feature extraction, ROI was stored as a three-dimensional cuttable image, and automatic extraction was performed using the MATLAB (R2011b, MathWorks) platform and the program developed in this research. The combined features included 851 first-order statistical features, shape, texture, and wavelet features.

### Statistical analysis

Demographic data of patients were divided into classified and numerical variables. Pearson’s Chi-square test or accurate Fisher’s test was used to compare classification variables between groups and the Mann–Whitney U test was applied for continuous variables. All statistical analyses were performed using R software version 3.6.3 (R Statistical Computing Foundation, Vienna, Austria) and X-Tile software version 3.6.1 (Yale University School of Medicine, New Haven, CT, USA). Extracted radiomics data were standardized using Z-score. The least absolute shrinkage and selection operator (LASSO) Cox regression model was used for data dimensionality reduction, feature selection, and radiological feature construction to select the most valuable predictive radiological features from tumor images. A nomogram was developed to determine survival outcomes based on radiological features, and a multivariate Cox regression risk model was established. A nomogram, also known as a nomograph, is a graphical method of evaluating complex functions. In this study, a nomogram was constructed using radiation features to assess patient survival. This method visually represents the value range of different variables and their contribution to the value of risk. The “glmnet” package in R software was used to select radiological characteristics consistent with the Cox proportional model, and the “survival” and “RMS” packages in R software were used to generate the multivariate Cox regression risk model, nomogram, and correction curve, respectively. The area under the receiver operating characteristic (ROC) curve (AUC) was used to evaluate the performance of the nomogram model. Using a linear combination of selected radiological features weighted by their respective coefficients, the radiomics score (rad-score) for each patient was calculated. The cut-off value of the rad-score was determined based on X-Tile software and used to classify patients into high- and low-risk groups.

## Results

**Fig. 1:**
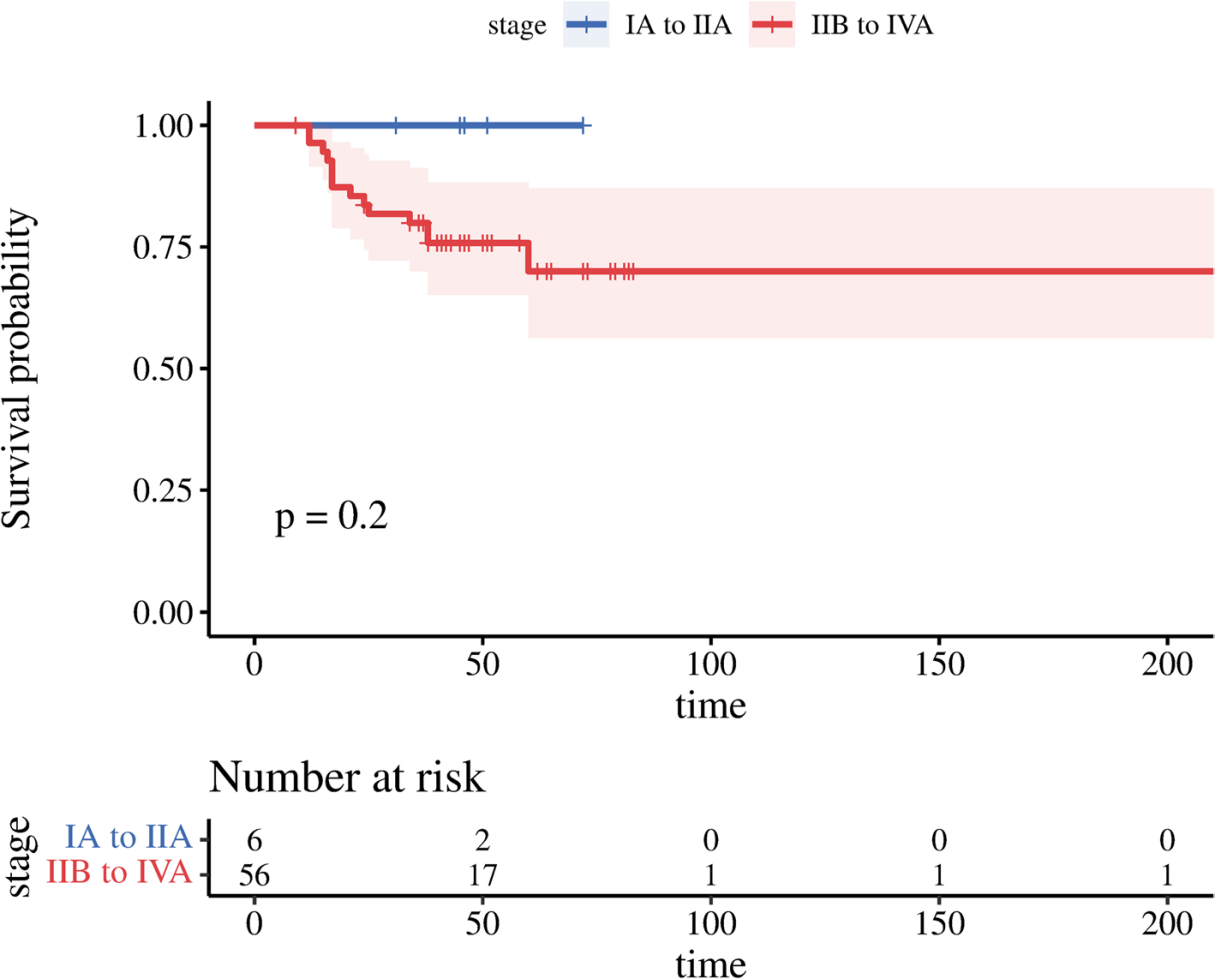
Kaplan–Meier survival plot of 62 enrolled patients

The clinical characteristics of the 62 enrolled patients are presented in Table 1. All of the 62 patients included in the study were female. The average patient age was 53.27 ± 8.29 years, 41 patients (66.1%) were 50 years old or above, while 21 patients (33.9%) were younger than 50. Regarding cancer stage, 47 patients (75.8%) were at stage IIB, while 4 patients (6.5%) were at stage IIIB. The remaining 10 patients (17.7%) were stage in another category. At the time of analysis, 48 patients (77.4%) were still alive. The median survival time was 24 weeks (95% confidence interval). and median follow-up time was 30 months (6–96 months). Kaplan-May’s survival plot is shown in Fig. 1.

**Table 1:** Demographic and baseline clinical data of study subjects

Characteristics	N (%)
Age (years)	
≥ 50	41 (66.1)
< 50	21 (33.9)
Stage	
IB	1 (1.6)
IIA	5 (8.1)
IIB	47 (75.7)
IIIA	4 (6.5)
IIIB	4 (6.5)
IVA	1 (1.6)
Risk group	
Low risk group	6 (9.6)
High risk group	56 (90.4)
Maximal diameter of tumor (cm)	
< 3	8 (12.8)
≥ 3 and < 4	14 (22.6)
≥ 4 and < 5	12 (19.5)
≥ 5	28 (45.1)
Lymph node status	
Negative	46 (74.2)
Positive	16 (25.8)

All 851 radiation features were extracted, including: shape (descriptors of 2D and 3D size and shape of the ROI, which were independent of the grayscale intensity distribution in the ROI and therefore calculated solely based on nonderived images and masks); first-order features describing the distribution of voxel intensity in the image region defined by the mask by common basic metrics; gray-level cooccurrence matrix (GLCM) features describing the second-order joint probability function of the image region constrained by the mask; gray-level dependence matrix (GLDM) features quantifying the gray correlation in the image; gray-level run-length matrix (GLRLM) elements, quantitatively defined as the length of consecutive pixels with the same gray scale value; gray-level size zone matrix (GLSZM) elements (the grayscale area in the quantized image); and neighboring gray tone difference matrix (NGTDM) elements quantifying the differences between the grayscale and average grayscale values of adjacent pixels within a certain distance.

**Fig. 2:**
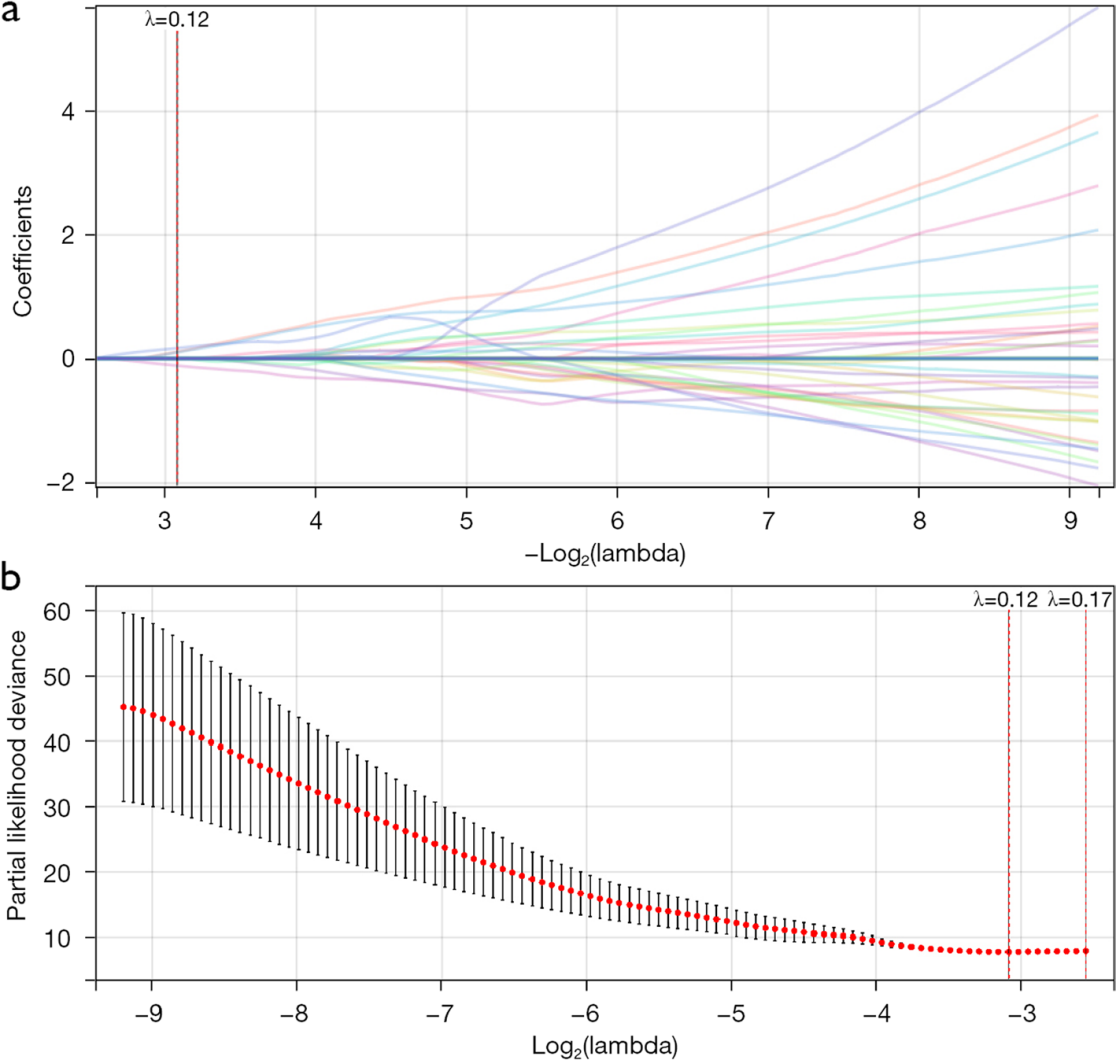
LASSO coefficient profiles of the 851 texture features. **a** Selection of radiomic features using the LASSO regression method. Optimal penalization coefficient lambda (λ = 0.12) in the LASSO model tuned using tenfold cross-validation and the minimum criterion (**b**) LASSO. LASSO, least absolute shrinkage and selection operator

Among the 851 radiomics features extracted from T1WI, T2WI, and DWI, potential features were selected based on their coefficients in the LASSO logistic regression model in the primary cohort (Fig. 2a). At λ = 0.12, the error of the model was minimum, and the characteristic number with nonzero coefficient was 4. High-throughput radiological features were reduced by LASSO regression (Fig. 2). Four radiological features were extracted for OS analysis, including short-range low gray emphasis (SRLGLE) of wavelet-HLL (H = high frequency band, L = low frequency band) firstorderMean (feature 1), wavelet-HLLfirstorderMedian (feature 2), wavelet-LLLgldmDependenceVariance (feature 3), and originalglemInverseVariance (feature 4).

**Fig. 3:**
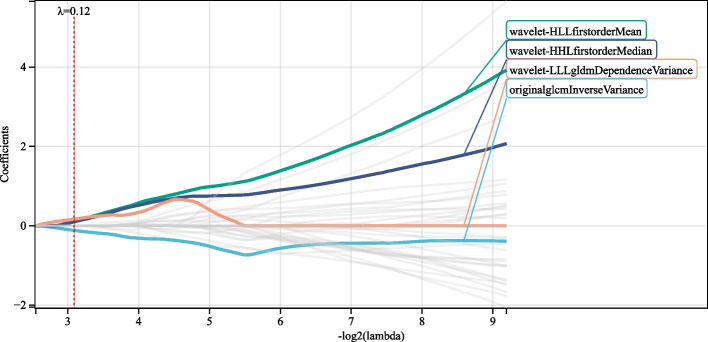
Prognostic radiomics features selected using the LASSO-Cox algorithm. LASSO, least absolute shrinkage and selection operator

The LASSO-Cox regression approach was used to select and screen 4 radiomics features (Fig. 3). The radiomics score was calculated according to the feature name and corresponding weight coefficient (Table 2). Features 1–4 represent radiomic features calculated via rad-score using the following formula [1]:

$$\mathrm{RiskScore}\;=\;-0.115376\;\times\;\mathrm{originalglcmInverseVariance}\;+\;0.107882\;\times\;\mathrm{wavelet}-\mathrm{HLLfirstorderMean}\;+\;0.099625\;\times\;\mathrm{wavelet}-\mathrm{HHLfirstorderMedian}\;+\;0.167106\;\;\times\;\mathrm{wavelet}-\mathrm{LLLgldmDependenceVariance}$$

**Table 2:** The 4 features screened based on the LASSO-Cox regression model

Feature	Coefficient
OriginalglcmInverseVariance	− 0.115376
Wavelet-HLLfirstorderMean	0.107882
Wavelet-HHLfirstorderMedian	0.099625
Wavelet-LLLgldmDependenceVariance	0.167106

*LASSO* least absolute shrinkage and selection operator

**Fig. 4:**
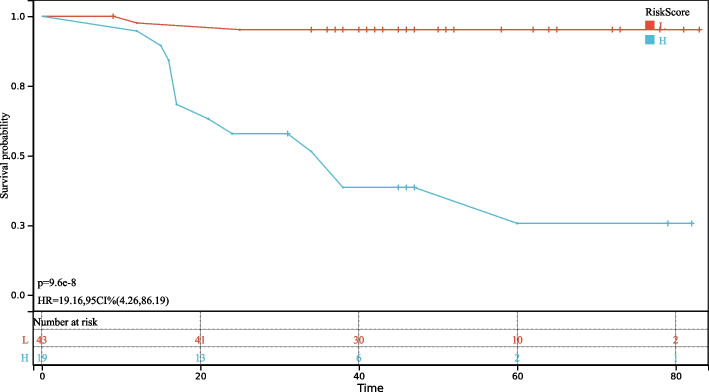
Survival curve of the high- and low-risk groups based on radiomics score classification. HR, hazard ratio; CI, confidence interval

**Fig. 5:**
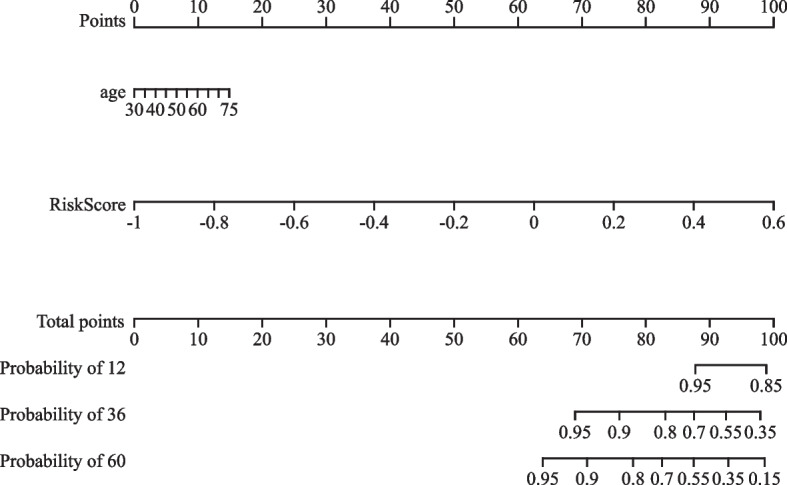
Nomogram of the combination of radiomic features

The patients in the study were stratified into two groups, based on their rad-score: high-risk (rad-score ≥  − 0.1) and low-risk (rad-score <  − 0.1) groups. The survival curves for these two groups are presented in Fig. 4 (*P* < 0.000). Additionally, a line diagram depicting the combined radiological features is presented in Fig. 5.

**Fig. 6:**
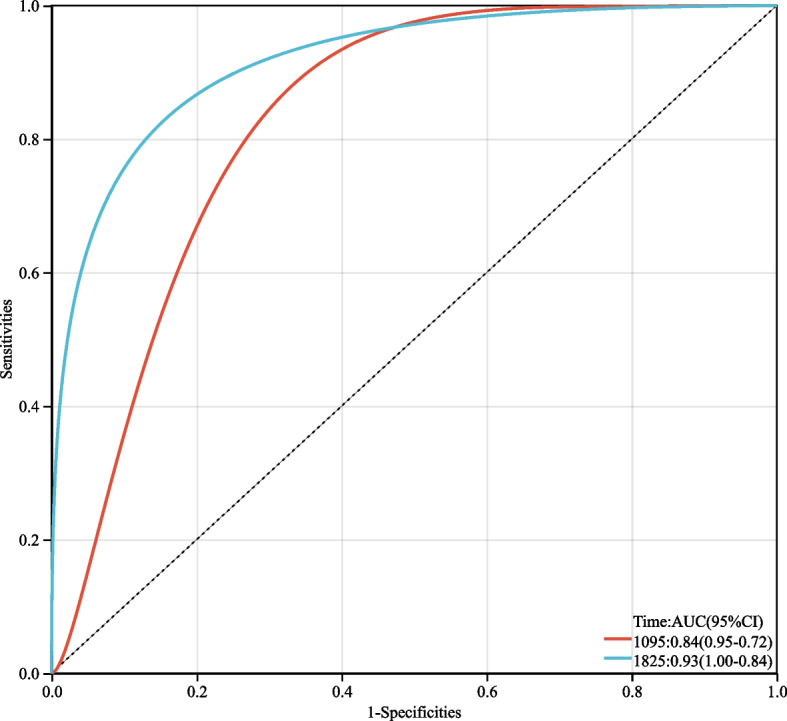
ROC curve of the model for prediction of patient OS at 3 and 5 years. AUC, area under the ROC curve; ROC, receiver operating characteristic; CI, confidence interval; OS, overall survival

Use AUC to evaluate the predictive ability of the model. As shown in Fig. 6. For 3-year OS prediction, AUC was 0.84 (95% confidence interval: 0.69–0.89), sensitivity was 0.81 (95% confidence interval: 0.76–0.86), specificity was 0.50 (95% confidence interval: 0.37, 0.63), accuracy was 0.74 (95% confidence interval: 0.70–0.79), 5-year OS prediction AUC was 0.93 (95% confidence interval: 0.85–0.98), sensitivity was 0.85 (95% confidence interval: 0.79–0.91), specificity was 0.59 (95% confidence interval: 0.47, 0.71), The accuracy is 0.81 (95% confidence interval: 0.78–0.86).

## Discussion

This study aimed to assess the potential prognostic value of MRI radiological features in cervical cancer patients who underwent surgery combined with radiotherapy and chemotherapy. Accurate prediction of disease progression after treatment is crucial in clinical practice, as it informs the selection of appropriate adjuvant therapy and helps improve patient outcomes [20]. However, disease progression can vary significantly among patients with similar clinical profiles, underscoring the need for reliable prognostic biomarkers.

MRI is known for its exceptional sensitivity to changes in biological tissue microstructure [21–23]. It enables noninvasive assessment of various tumor characteristics, including cell density and hypoxia, which are indicators of tumor heterogeneity, such as necrosis and increased cell density. Based on these characteristics, we hypothesized that MRI radiological features could be used effectively to predict tumor progression [24, 25]. Initially used to distinguish between tumor and normal cervical tissues, MRI has evolved as a promising diagnostic tool for detecting pelvic LNM and monitoring treatment response in patients with cervical cancer [26, 27]. With the expansion of its clinical applicability, an MRI-based candidate index has recently been proposed for predicting clinical outcomes in cervical cancer patients. However, the results obtained from various studies have been highly variable. For instance, some studies have reported higher recurrence rates associated with higher ADC values [28], while others have shown that lower ADC values are better predictors of recurrence than well-established prognostic factors such as parauterine invasion or lymphovascular invasion (LVI) [29, 30]. Although radiomics analyses are becoming increasingly mature, there are some technical limitations. Many radiomics features, increasingly extracted via radiomics software packages, are sensitive to variations based on image acquisition, reconstruction, and processing procedures; therefore, different feature-generation hyperparameters, fusion techniques, and segmentation methods may produce variable radiomics features. The development and application of image fusion and other technologies will effectively improve the effect of image preprocessing [31].

This variability in results highlights the need for further studies to establish the prognostic value of MRI-based candidate indices in cervical cancer. It is crucial to determine the most reliable MRI-based parameters for predicting clinical outcomes, as this could help identify patients at high risk of recurrence, guide treatment decisions, and improve overall patient outcomes. Nonetheless, the growing body of research on the use of MRI in cervical cancer holds great promise for improving diagnosis, monitoring, and management of this complex disease.

Prior research has demonstrated the potential utility of radiology in predicting survival of cervical cancer patients, with reported accuracy higher than that of traditional clinical factors [32–35]. However, the sample sizes of these studies were relatively small and did not focus on early cervical cancer cases treated with surgery. As such, the clinical relevance of these findings is limited, given that patient treatment is a strong predictor of survival. Moreover, due to the lack of clinical consensus on the use of MRI for this indication, there is a need for further research in this area.

Our study provides reliable evidence for the application of imaging in prognosis and offers key insights for further research. Our radiomics scores, based on LASSO-Cox, demonstrated good predictive performance for estimating OS. These findings may assist in identifying patients with poor survival prognosis who may benefit from more comprehensive investigation and active treatment options, aiding clinical decision-making and improving patient outcomes. Radiomics offers a promising approach to extract useful imaging features from medical images noninvasively, providing potential diagnostic, therapeutic, and prognostic information [26, 36–42].

The prediction model developed in this study aimed to predict the 3- and 5-year OS of patients with early-stage cervical cancer who underwent surgery. The model’s performance was evaluated using the AUC. The ROC curve analysis showed that the 3-year OS prediction model had an AUC value of 0.84, indicating moderate discrimination ability in distinguishing between patients who survive and those who do not survive at 3 years. Similarly, the 5-year OS prediction model had an AUC value of 0.93, indicating excellent discrimination ability in distinguishing between patients who survive and those who do not survive at 5 years. These findings suggest that the prediction model has reasonably accurate predictive ability, which may have clinical utility in guiding treatment decisions and patient counseling. The derived model seems to predict the outcome more accurately than the FIGO staging system. Although this nomogram must be externally validated before it can be applied, it may be valuable in terms of choosing adjunctive treatment, counseling patients, and planning clinical trials. However, it is important to note that the accuracy of the model may vary depending on the specific patient population and clinical setting in which it is used.

MRI radiological models have shown to be useful for predicting preoperative LNM and LVI status in cervical cancer patients [43, 44]. In addition, radiological features derived from MRI scans can be effectively used to predict survival of early-stage cervical cancer patients [45, 46]. In this study, we used the rad-score to classify patients into high- and low-risk groups. Our findings showed that higher rad-scores were associated with poorer OS, suggesting that low-risk patients may undergo unnecessary radical hysterectomy, while systemic adjuvant therapy may be more beneficial for patients with a higher risk of recurrence and metastasis. These results are a critical step towards tailoring treatments to specific clinical and radiological characteristics of high- and low-risk patients with early-stage cervical cancer.

Among the 851 imaging signs, we found that 4 T1W images were predictive of OS. This highlights the potential benefits of radiomics methods in mining high-dimensional information that is difficult to interpret, and the advantage of using T1W images over T2W and DWI images. While FIGO staging and LNM are commonly used in prognostic assessment of cervical cancer, their predictive power is limited [47–50]. Previous studies have also reported the efficacy of ADC histograms in assessing the prognosis of cervical cancer, but the results are varied. Radiomics is an emerging quantitative method that aims to use advanced technologies for noninvasive capture of tumor phenotypic characteristics, which are more likely to facilitate prediction of clinical prognosis of patients before treatment.

Our study provides valuable insights into the potential clinical utility of MRI radiological features as a prognostic tool for cervical cancer patients receiving combined treatment. The results suggest that these features could help identify patients at high risk of disease progression, enabling clinicians to tailor treatment regimens accordingly. However, the study had some limitations, including the small sample size of the training and validation sets. Further external prospective verification with larger sample sizes is needed. Additionally, our study only focused on radiomics analysis of ADC maps, and further in-depth investigations of multiparameter fusion techniques, including dynamic enhancement of tumor blood supply-related parameters, are warranted.

In conclusion, our study demonstrates the clinical value of the radiomics model established using the ADC parameter map for predicting long-term OS in cervical cancer patients. MRI-based radiological features have the potential to serve as a valuable prognostic tool for clinicians in managing patients with cervical cancer undergoing surgery in conjunction with radiotherapy and chemotherapy.

## Data Availability

The datasets used or analysed during the current study are available from the corresponding author on reasonable request.

## References

[1] Mahantshetty U, Lavanya G, Grover S (2021). Incidence, Treatment and Outcomes of Cervical Cancer in Low- and Middle-income Countries. Clin Oncol (R Coll Radiol).

[2] Arbyn M, Weiderpass E, Bruni L (2020). Estimates of incidence and mortality of cervical cancer in 2018: a worldwide analysis. Lancet Glob Health.

[3] Ginsburg OM (2013). Breast and cervical cancer control in low and middle-income countries: Human rights meet sound health policy. J Cancer Policy.

[4] Lee JT, Lee YH, Chang YP (2022). Mindfulness Stress Management for Female Cancer Survivors Facing the Uncertainty of Disease Progression: A Randomized Controlled Study. Int J Environ Res Public Health.

[5] Johnson CA, James D, Marzan A (2019). Cervical Cancer: An Overview of Pathophysiology and Management. Semin Oncol Nurs.

[6] Wipperman J, Neil T, Williams T (2018). Cervical Cancer: Evaluation and Management. Am Fam Physician.

[7] Chao X, Song X, Wu H (2021). Selection of Treatment Regimens for Recurrent Cervical Cancer. Front Oncol.

[8] Tewari KS, Monk BJ (2019). Evidence-Based Treatment Paradigms for Management of Invasive Cervical Carcinoma. J Clin Oncol.

[9] Lee SI, Atri M (2019). 2018 FIGO Staging System for Uterine Cervical Cancer: Enter Cross-sectional Imaging. Radiology.

[10] Wu M, Wu J, Huang L (2022). Comparison of contrast-enhanced ultrasonography and magnetic resonance imaging in the evaluation of tumor size and local invasion of surgically treated cervical cancer. Abdom Radiol (NY).

[11] Stukan M, Buderath P, Szulczyński B (2021). Accuracy of Ultrasonography and Magnetic Resonance Imaging for Preoperative Staging of Cervical Cancer-Analysis of Patients from the Prospective Study on Total Mesometrial Resection. Diagnostics (Basel).

[12] Woo S, Atun R, Ward ZJ (2020). Diagnostic performance of conventional and advanced imaging modalities for assessing newly diagnosed cervical cancer: systematic review and meta-analysis. Eur Radiol.

[13] Gui B, Autorino R, Miccò M (2021). Pretreatment MRI Radiomics Based Response Prediction Model in Locally Advanced Cervical Cancer. Diagnostics (Basel).

[14] Qu JR, Qin L, Li X (2018). Predicting Parametrial Invasion in Cervical Carcinoma (Stages IB1, IB2, and IIA): Diagnostic Accuracy of T2-Weighted Imaging Combined With DWI at 3 T. AJR Am J Roentgenol.

[15] Liu Z, Wang S, Dong D (2019). The Applications of Radiomics in Precision Diagnosis and Treatment of Oncology: Opportunities and Challenges. Theranostics.

[16] Tagliafico AS, Piana M, Schenone D (2020). Overview of radiomics in breast cancer diagnosis and prognostication. Breast.

[17] Ai Y, Zhu H, Xie C (2020). Radiomics in cervical cancer: Current applications and future potential. Crit Rev Oncol Hematol.

[18] Deng X, Liu M, Sun J (2021). Feasibility of MRI-based radiomics features for predicting lymph node metastases and VEGF expression in cervical cancer. Eur J Radiol.

[19] Li Z, Li H, Wang S (2019). MR-Based Radiomics Nomogram of Cervical Cancer in Prediction of the Lymph-Vascular Space Invasion preoperatively. J Magn Reson Imaging.

[20] Poldrack RA, Huckins G, Varoquaux G (2020). Establishment of Best Practices for Evidence for Prediction: A Review. JAMA Psychiat.

[21] Martinez-Heras E, Grussu F, Prados F (2021). Diffusion-Weighted Imaging: Recent Advances and Applications. Semin Ultrasound CT MR.

[22] Schilling KG, Janve V, Gao Y (2018). Histological validation of diffusion MRI fiber orientation distributions and dispersion. Neuroimage.

[23] Wichtmann BD, Zöllner FG, Attenberger UI (2021). Multiparametric MRI in the Diagnosis of Prostate Cancer: Physical Foundations, Limitations, and Prospective Advances of Diffusion-Weighted MRI. Rofo.

[24] Bera K, Braman N, Gupta A (2022). Predicting cancer outcomes with radiomics and artificial intelligence in radiology. Nat Rev Clin Oncol.

[25] Majumder A, Sen D (2021). Artificial intelligence in cancer diagnostics and therapy: current perspectives. Indian J Cancer.

[26] Haldorsen IS, Lura N, Blaakær J (2019). What Is the Role of Imaging at Primary Diagnostic Work-Up in Uterine Cervical Cancer?. Curr Oncol Rep.

[27] Haldorsen IS, Salvesen HB (2016). What Is the Best Preoperative Imaging for Endometrial Cancer?. Curr Oncol Rep.

[28] Marias K (2021). The Constantly Evolving Role of Medical Image Processing in Oncology: From Traditional Medical Image Processing to Imaging Biomarkers and Radiomics. J Imaging.

[29] Yan BC, Xiao ML, Li Y, et al. The diagnostic performance of ADC value for tumor grade, deep myometrial invasion and lymphovascular space invasion in endometrial cancer: a meta-analysis. Acta Radiol. 2019. 10.1177/0284185119841988. [Epub ahead of print]. 10.1177/0284185119841988 31042066

[30] Zhou P, Jin C, Lu J (2021). The Value of Nomograms in Pre-Operative Prediction of Lymphovascular Invasion in Primary Breast Cancer Undergoing Modified Radical Surgery: Based on Multiparametric Ultrasound and Clinicopathologic Indicators. Ultrasound Med Biol.

[31] Salmanpour MR, Hosseinzadeh M, Rezaeijo SM (2023). Fusion-based tensor radiomics using reproducible features: Application to survival prediction in head and neck cancer[J]. Comput Methods Programs Biomed.

[32] Liu S, Li R, Liu Q (2022). Radiomics model of 18F-FDG PET/CT imaging for predicting disease-free survival of early-stage uterine cervical squamous cancer. Cancer Biomark.

[33] Li H, Zhu M, Jian L (2021). Radiomic Score as a Potential Imaging Biomarker for Predicting Survival in Patients With Cervical Cancer. Front Oncol.

[34] Zheng RR, Cai MT, Lan L (2022). An MRI-based radiomics signature and clinical characteristics for survival prediction in early-stage cervical cancer. Br J Radiol.

[35] Zhang X, Zhao J, Zhang Q (2022). MRI-based radiomics value for predicting the survival of patients with locally advanced cervical squamous cell cancer treated with concurrent chemoradiotherapy. Cancer Imaging.

[36] Lambin P, Leijenaar RTH, Deist TM (2017). Radiomics: the bridge between medical imaging and personalized medicine. Nat Rev Clin Oncol.

[37] Scapicchio C, Gabelloni M, Barucci A (2021). A deep look into radiomics. Radiol Med.

[38] Gatta R, Depeursinge A, Ratib O (2020). Integrating radiomics into holomics for personalised oncology: from algorithms to bedside. Eur Radiol Exp.

[39] Qi YX, Liu K, Yin J (2018). Evaluation of short- and long-term efficacy of chemoradiotherapy for advanced cervical cancer using HSP70 protein combined with multimodal MRI. J Cell Biochem.

[40] Dappa E, Elger T, Hasenburg A (2017). The value of advanced MRI techniques in the assessment of cervical cancer: a review. Insights Imaging.

[41] Lura N, Wagner-Larsen KS, Forsse D (2022). What MRI-based tumor size measurement is best for predicting long-term survival in uterine cervical cancer?. Insights Imaging.

[42] Ho JC, Allen PK, Bhosale PR (2017). Diffusion-Weighted Magnetic Resonance Imaging as a Predictor of Outcome in Cervical Cancer After Chemoradiation. Int J Radiat Oncol Biol Phys.

[43] Fields EC, Weiss E (2016). A practical review of magnetic resonance imaging for the evaluation and management of cervical cancer. Radiat Oncol.

[44] Fang J, Zhang B, Wang S (2020). Association of MRI-derived radiomic biomarker with disease-free survival in patients with early-stage cervical cancer. Theranostics.

[45] Xiao M, Ma F, Li Y (2020). Multiparametric MRI-Based Radiomics Nomogram for Predicting Lymph Node Metastasis in Early-Stage Cervical Cancer. J Magn Reson Imaging.

[46] Wang T, Gao T, Yang J (2019). Preoperative prediction of pelvic lymph nodes metastasis in early-stage cervical cancer using radiomics nomogram developed based on T2-weighted MRI and diffusion-weighted imaging. Eur J Radiol.

[47] Widschwendter P, Janni W, Scholz C (2019). Prognostic factors for and pattern of lymph-node involvement in patients with operable cervical cancer. Arch Gynecol Obstet.

[48] Huang H, Liu Q, Zhu L (2019). Prognostic Value of Preoperative Systemic Immune-Inflammation Index in Patients with Cervical Cancer. Sci Rep.

[49] Yan DD, Tang Q, Chen JH (2019). Prognostic value of the 2018 FIGO staging system for cervical cancer patients with surgical risk factors. Cancer Manag Res.

[50] Kwon J, Eom KY, Kim YS (2018). The Prognostic Impact of the Number of Metastatic Lymph Nodes and a New Prognostic Scoring System for Recurrence in Early-Stage Cervical Cancer with High Risk Factors: A Multicenter Cohort Study (KROG 15–04). Cancer Res Treat.

